# P-1960. Safety and Pharmacokinetics of Long-acting SARS-CoV-2 Antibodies Tixagevimab/Cilgavimab (AZD7442) are Consistent after Repeat Dosing: Results from the PROVENT Substudy

**DOI:** 10.1093/ofid/ofae631.2119

**Published:** 2025-01-29

**Authors:** Andrew Ustianowski, Myron J Levin, Stéphane De Wit, Odile Launay, Hilde Bollen, Tommy Rampling, James G Sullivan, Mark Vishnepolsky, Priyantha Wijewardane, Audrey Sharbaugh, Rohini Beavon, Jesse Thissen, Lauren Hirao, Vitalina Dzutseva, Seth Seegobin, Katie Streicher, Alexandre Kiazand, Mark T Esser, Taylor Cohen, Lee-Jah Chang, John L Perez

**Affiliations:** North Manchester General Hospital, Manchester, England, United Kingdom; University of Colorado Denver School of Medicine, Aurora, Colorado; CHU St-Pierre, Brussels, Brussels Hoofdstedelijk Gewest, Belgium; Université Paris Cité; Inserm F-CRIN, I-REIVAC; Assistance Publique Hôpitaux de Paris, Paris, Ile-de-France, France; Anima Research Center, Alken, Limburg, Belgium; University College London Hospitals NHS Foundation Trust, London, England, United Kingdom; Parkway Medical Center, Birmingham, Alabama; Kidney Specialists of Southern Nevada; DaVita Clinical Research, Las Vegas, Nevada; Baptist Health Center for Clinical Research, Little Rock, Arkansas; AstraZeneca, Durham, North Carolina; AstraZeneca, Durham, North Carolina; AstraZeneca, Durham, North Carolina; AstraZeneca, Durham, North Carolina; AstraZeneca, Durham, North Carolina; AstraZeneca, Durham, North Carolina; AstraZeneca, Durham, North Carolina; AstraZeneca, Durham, North Carolina; AstraZeneca, Durham, North Carolina; AstraZeneca, Durham, North Carolina; AstraZeneca, Durham, North Carolina; AstraZeneca, Durham, North Carolina

## Abstract

**Background:**

The SARS-CoV-2 antibody combination tixagevimab/cilgavimab (AZD7442) was efficacious in preventing COVID-19 over 6 months following a single 300 mg intramuscular (IM) dose in the PROVENT study. Participants included those that: (a) were immunocompromised and/or at increased risk for inadequate response to COVID-19 vaccination, or (b) were at increased severe COVID-19 risk. Following PROVENT, emergence of Omicron variants with reduced AZD7442 susceptibility prompted increase in the authorized dose and dosing frequency. Here, we report safety and pharmacokinetics (PK) of repeated doses of AZD7442 300–600 mg in participants who opted to continue in the PROVENT substudy.

**Figure:**
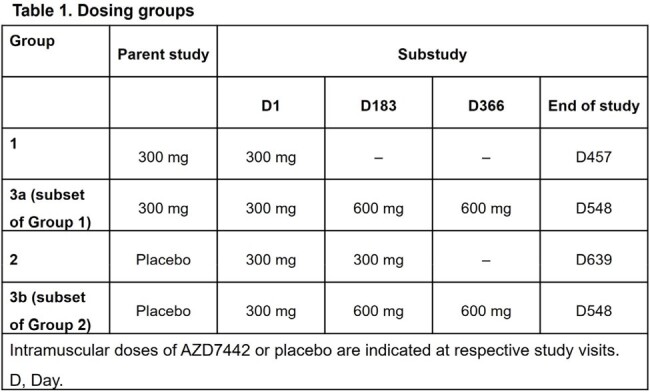

**Methods:**

Participants who entered the PROVENT open-label sub-study (NCT04625725) were assigned initially to 2 groups: Group 1 (n=234) received 1x AZD7442 300 mg in the parent study, then 1x 300 mg ∼10–14 months later (substudy Day 1). Group 2 (n=119) received placebo in the parent study, then 2x AZD7442 300 mg doses in the substudy. Group 3a (n=76) was a subset of Group 1 who received 2x 300 mg then 2x 600 mg doses. Group 3b (n=74) was a subset of Group 2 who received 1x 300 mg then 2x 600 mg doses (Table 1). The primary endpoint was safety; PK and antibodies to AZD7442 were also assessed.

**Figure:**
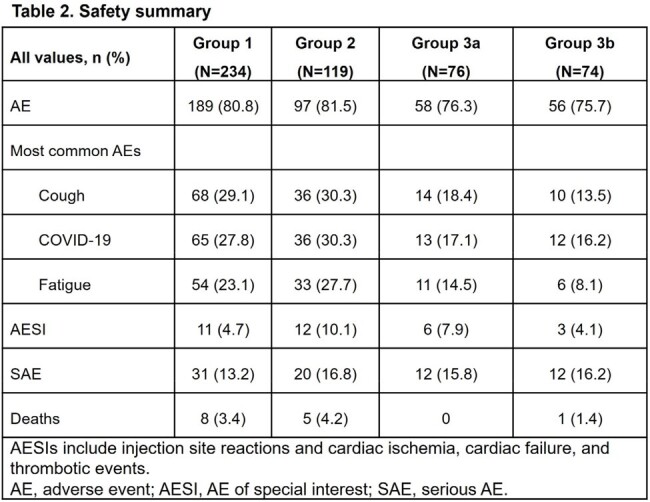

**Results:**

Baseline characteristics were overall similar across the groups. Adverse events (AEs) were reported in 75.7–81.5% and serious AEs in 13.2–16.8% of participants across the groups (Table 2). There were 14 deaths: 8 (3.4%) in Group 1, 5 (4.2%) in Group 2, and 1 (1.4%) in Group 3b. There was no clinically meaningful increase of AEs due to repeat doses of AZD7442 and no deaths were considered related to AZD7442. AZD7442 PK was consistent following redosing and between groups (Figure 1). The percentage of participants positive for treatment-emergent antibodies to AZD7442 was 10.5% (Group 1), 5.2% (Group 2), 10.7% (Group 3a), and 4.1% (Group 3b).

**Figure:**
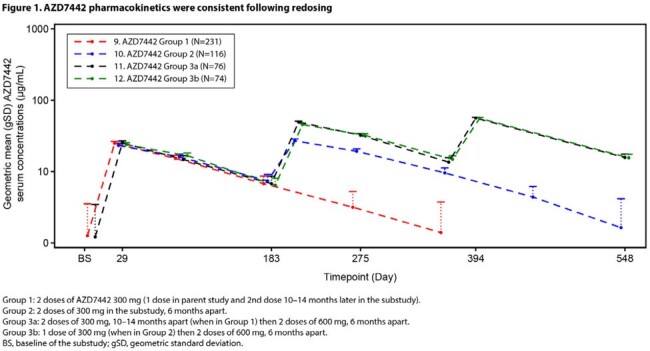

**Conclusion:**

Safety and PK of AZD7442 were consistent after repeat dosing, regardless of whether participants initially received AZD7442 or placebo. These results will support future development of long-acting antibodies for pre-exposure prophylaxis against COVID-19.

**Disclosures:**

Andrew Ustianowski, MD, PhD, AstraZeneca: Honoraria|Gilead: Honoraria|GSK: Honoraria|GSK: Speaker fees|Janssen: Honoraria|Janssen: Speaker fees|Merck: Honoraria|Merck: Speaker fees, Advisory Board|Pfiizer: Advisory Board|Sanofi: Honoraria|Sanofi: Speaker fees|ViiV Healthcare/GSK: Advisory Board Myron J. Levin, MD, CSL Seqirus USA: Advisor/Consultant|Curevo: Advisory Board|GSK: Grant/Research Support|GSK: Advisory Board|Moderna: Advisory Board|Pfizer: Advisory Board Stéphane De Wit, MD, AstraZeneca: Honoraria|AstraZeneca: Speaker fees|Gilead: Honoraria|Gilead: Speaker fees, Advisory Board|GSK: Honoraria|GSK: Speaker fees|Janssen: Honoraria|Janssen: Speaker fees|Merck: Honoraria|Merck: Speaker fees, Advisory Board|Pfizer: Advisory Board|Sanofi: Honoraria|Sanofi: Speaker fees|ViiV Healthcare/GSK: Advisory Board Odile Launay, MD, PhD, AstraZeneca: Principal investigator for the AstraZeneca-sponsored PROVENT study in France|AstraZeneca and other pharmaceutical companies: Honoraria|AstraZeneca and other pharmaceutical companies: Speaker fees Audrey Sharbaugh, PhD, AstraZeneca: Employee, holds or may hold stock Rohini Beavon, PhD, AstraZeneca: Employee, holds or may hold stock Jesse Thissen, MSc, AstraZeneca: Employee, holds or may hold stock Lauren Hirao, PhD, AstraZeneca: Employee, holds or may hold stock Vitalina Dzutseva, MD, PhD, AstraZeneca: Employee, holds or may hold stock Seth Seegobin, PhD, AstraZeneca: Employee, holds or may hold stock Katie Streicher, PhD, AstraZeneca: Employee of AstraZeneca and may own AstraZeneca stock or stock options. Alexandre Kiazand, MD, AstraZeneca: Employee, holds or may hold stock Mark T. Esser, PhD, AstraZeneca: Employee, holds or may hold stock Taylor Cohen, PhD, AstraZeneca: Employee, holds or may hold stock Lee-Jah Chang, MD, AstraZeneca: Employee of AstraZeneca John L. Perez, MD, AstraZeneca: Employee, holds or may hold stock

